# The Ambulance in Peace and War

**Published:** 1895-07-20

**Authors:** 


### The Institutional Workshop.

### THE AMBULANCE IN PEACE AND WAR.

### Paris Ambulance Service.

It is somewhat surprising to those accustomed to the prompt, ready, and efficient aid rendered in cases of accident in the streets of London by a well-organised ambulance service, to find how entirely lacking in a similar organisation is Paris, proudly called by its citizens "La capitale du monde." Strong representations have been made to the responsible authorities, but these do not seem to be inclined to bestir themselves in the matter. There are three bodies which divide between them such responsibilities: the "Préfecture de la Seine," the "Préfecture de police," and "L'Assistance publique." Of the first-named it is said that its ambulances are seldom in time at any serious accident, while the second possesses no adequate means of treating severe injuries, or even of rendering ordinary first aid; and the last, relying on the services of the other two, does not possess "a single carriage or a single stretcher" capable of being sent out from the hospitals in the case of neighbouring accidents. As a proof of the lack of proper administration in cases of street accidents, in a late number of *L'Assistance* will be found a letter from M. le Professeur Terrier, giving particulars of a case of a carter whose leg was badly crushed in a street accident in Paris, and who ultimately died from the effects of the hæmorrhage ensuing upon the want of necessary "first-aid" succour, as an example of "l'absence absolue de secours chirurgicaux pour les blessés relevés sur la voie publique dans la ville de Paris." Such a condition of things is certainly very serious at the present day, in one of the first cities of the world, and urgent need undoubtedly exists for a thorough reorganisation of the ambulance system if Paris is to bring herself in this respect into line with London, Vienna, and other large and important capitals.

### The Hospital Corps in Madagascar.

The furnishing of hospital munitions for the Madagascar expedition has been carried out on a very thorough scale. The hospital corps consists of ambulances Nos. 1, 2, and 3, and of field hospitals. Ambulance No. 1, for infantry divisions, is provided with materials for nearly 7,000 dressings, and with 132 stretchers. No. 2 is supplied with dressings and necessaries for at least 800 wounded; the medical supplies are transported partly in cases and partly in bulk, and the stretchers number 22. Each of these two ambulances will be provided with a small camp chapel. It is the third ambulance which will bear the brunt of service in the war. It is intended for mountain work, and all supplies can be carried on baggage mules and packed in cases and canteens. The weight of the supplies to be carried is about 908 kilogrammes, and comprise some 1,200 dressings. The transport of the sick and wounded will be also by means of baggage mules. Each field hospital (there are four to each division) is fitted for the treatment of 100 sick and wounded for three months. Great care has been exercised in regard to the health of the men selected for the expedition, and the medical examinations have been searching and severe. In enforcing such extreme care the Minister for War has, no doubt, acted in the wisest manner. In addition to the necessaries carried by the ambulances, it has been wisely decreed that each man, officer and private alike, shall carry a small packet of indispensable dressings and bandages. These were not, however, delivered to the regiments until the actual moment of disembarkation at Madagascar.

